# Multiple inpatient admissions for cannabis-induced psychotic disorder - sociodemographic, clinical and treatment evaluation

**DOI:** 10.1192/j.eurpsy.2022.2046

**Published:** 2022-09-01

**Authors:** A.S. Machado, A. Elias De Sousa, F. Andrade, M. Vieira-Coelho

**Affiliations:** 1 FMUP, Departamento De Neurociências Clínicas E Saúde Mental, Porto, Portugal; 2 Centro Hospitalar Universitário São João, Serviço De Psiquiatria, Porto, Portugal

**Keywords:** inpatient treatment, clinical biomarkers, Cannabis, Psychosis

## Abstract

**Introduction:**

Current evidence contradicts the idea that cannabis-induced psychotic disorder (CIPD) has an overall benign prognosis, with up to half of these patients being with a schizophrenia spectrum disorder later in life.

**Objectives:**

To characterize sociodemographic and clinical characteristics and treatment plan of inpatients with multiple admissions for CIPD over a one-year period, compared to those with a single admission.

**Methods:**

Retrospective observational study of inpatient episodes with CIPD between january 1st 2018 and september 30th 2021 in a tertiary psychiatric inpatient unit. Statistical analysis was performed using SPSS software, version 27.0.

**Results:**

Our sample included 80 inpatients, 15 (18.8%) with multiple admissions for CIPE within one year period and 65 (81.3%) with a single admission. The multiple admissions group had a median of 1 ±0,915 admissions within the same year. Being readmitted for CIPE was associated with outpatient compulsory treatment at discharge (OR 3,01 (95% CI 1,27-7,18, p=0,034). These patients had 3.14 higher odds of future admissions to psychiatry unit (CI 95% 1.70-5.78, p<0.001). We found no statistically significant differences regarding the sociodemographic and clinical characteristics, daily vs. occasional use of cannabis in patients with multiple admissions for CIPE.

**Conclusions:**

Patients with multiple admissions for CIPD tend to have more relapses and require assertive treatment measures. However, they did not differ regarding the sociodemographic and clinical characteristics studied from patients with single admissions. This suggests that additional assessment of these patients might be important to predict the course of the disease.

**Disclosure:**

No significant relationships.

